# Prevention of Stricture after Endoscopic Submucosal Dissection for Superficial Esophageal Cancer: A Review of the Literature

**DOI:** 10.3390/jcm10010020

**Published:** 2020-12-23

**Authors:** Takuto Hikichi, Jun Nakamura, Mika Takasumi, Minami Hashimoto, Tsunetaka Kato, Ryoichiro Kobashi, Tadayuki Takagi, Rei Suzuki, Mitsuru Sugimoto, Yuki Sato, Hiroki Irie, Yoshinori Okubo, Masao Kobayakawa, Hiromasa Ohira

**Affiliations:** 1 Department of Endoscopy, Fukushima Medical University Hospital, Fukushima-City 960-1295, Fukushima, Japan; junn7971@fmu.ac.jp (J.N.); mi-hashi@fmu.ac.jp (M.H.); tsune-k@fmu.ac.jp (T.K.); yoshi-o@fmu.ac.jp (Y.O.); mkobaya@fmu.ac.jp (M.K.); 2 Department of Gastroenterology, Fukushima Medical University School of Medicine, Fukushima-City 960-1295, Fukushima, Japan; paper@fmu.ac.jp (M.T.); rkobashi@fmu.ac.jp (R.K.); daccho@fmu.ac.jp (T.T.); subaru@fmu.ac.jp (R.S.); kita335@fmu.ac.jp (M.S.); dorcus@fmu.ac.jp (Y.S.); hirokiri@fmu.ac.jp (H.I.); h-ohira@fmu.ac.jp (H.O.); 3 Department of Medical Research Center, Fukushima Medical University, Fukushima-City 960-1295, Fukushima, Japan

**Keywords:** endoscopic submucosal dissection, esophageal cancer, prevention, stenosis, stricture

## Abstract

Endoscopic resection has been the standard treatment for intramucosal esophageal cancers (ECs) because of the low risk of lymph node metastases in the lesions. In recent years, endoscopic submucosal dissection (ESD), which can resect large ECs, has been performed. However, the risk of esophageal stricture after ESD is high when the mucosal defect caused by the treatment exceeds 3/4 of the circumference of the lumen. Despite the subsequent high risk of luminal stricture, ESD has been performed even in cases of circumferential EC. In such cases, it is necessary to take measures to prevent stricture. Therefore, in this review, we aimed to clarify the current status of stricture prevention methods after esophageal ESD based on previous literature. Although various prophylactic methods have been reported to have stricture-preventing effects, steroid injection therapy and oral steroid administration are mainstream. However, in cases of circumferential EC, both steroid injection therapy and oral steroid administration cannot effectively prevent luminal stricture. To solve this issue, clinical applications, such as tissue shielding methods with polyglycolic acid sheet, autologous oral mucosal epithelial sheet transplantation, and stent placement, have been developed. However, effective prophylaxis of post-ESD mucosal defects of the esophagus is still unclear. Therefore, further studies in this research field are needed.

## 1. Introduction

Endoscopic resection (ER) is the standard treatment for intramucosal esophageal cancers (ECs) [1]. Nowadays, endoscopic submucosal dissection (ESD) has been developed. It enables a reliable en bloc resection of large lesions and, accordingly, a favorable prognosis has been reported [2,3,4,5,6]. On the other hand, esophageal stricture after ESD has become a severe issue [7,8,9,10]; it reduces oral intake and requires dietary restrictions, leading to malnutrition and poor quality of life for patients. Traditionally, endoscopic balloon dilation (EBD) is performed to treat the postoperative stricture of ESD, but serious adverse events such as perforation may occur [11,12]. Moreover, frequent and long-term EBD imposes financial and psychological stress on patients. There is now a consensus that “mucosal defects of more than 3/4 of the lumen circumference are predictive factors of stricture after esophageal ESD” [13]. The methods of stricture prevention after esophageal ESD have been reported, as shown in Table 1. In this review, we aimed to clarify the current status of stricture prevention methods after esophageal ESD based on previous literature.

## 2. Prophylactic EBD

Inoue et al. [14] performed prophylactic EBD with manual air infusion using an 18–20 mm diameter balloon from one to three days and every day for the first week in patients after ESD of a total mucosal defect circumference (MDC); the circulation rate of the mucosal defect in the esophageal lumen after ER is defined as MDC. The median number of EBDs was 35.5, and the median duration was 100 days. Ezoe et al. [15] reported prophylactic EBD among patients with more than 3/4 MDC after EMR/ESD. Prophylactic EBD was initiated using a balloon with a diameter of 18–20 mm within one week of ER and continued once a week until the mucosal defect was closed. The incidence of stricture after prophylactic EBD was significantly lower than that without prophylactic treatments (59% vs. 92%, respectively), and the EBD duration required to improve the stricture was significantly shorter (29 days vs. 78 days, respectively). Yamaguchi et al. [16] performed prophylactic EBD twice a week for eight weeks, initiated three days after ESD in patients with more than 3/4 MDC. However, the incidence of stricture in patients with prophylactic EBD was significantly higher than with oral steroids (31.8% vs. 5.3%, respectively).

Li et al. [17] devised a self-help inflatable balloon, which was 18 mm in diameter and was inflated with 35 mL of air. The balloon was inserted intranasally four days after ESD, and patients inflated the balloon on their own 4–5 times a day for 15–20 min each time until the mucosal defect was closed. Among eight patients with total MDC, the incidence of stricture was 12.5%, and three sessions of EBDs were required for one patient to improve the stricture. Adverse events such as pharyngeal and nasal pain occurred, but no perforation was observed.

In summary, prophylactic EBD requires multiple endoscopic sessions and is inferior to oral steroids in preventing stricture after esophageal ESD. However, a self-help inflatable balloon seems to be an interesting device.

## 3. Steroid Therapy

Table 2 summarizes the comparative studies of steroid therapy in the prevention of stricture after esophageal ESD, mainly compared with no therapy or prophylactic EBD [18]. We also present the results of steroid-based stricture prophylaxis for non-total MDC (Table 3) and total MDC after esophageal ESD (Table 4) [19,20].

## 4. Steroid Injection Therapy

**Figure 1:**
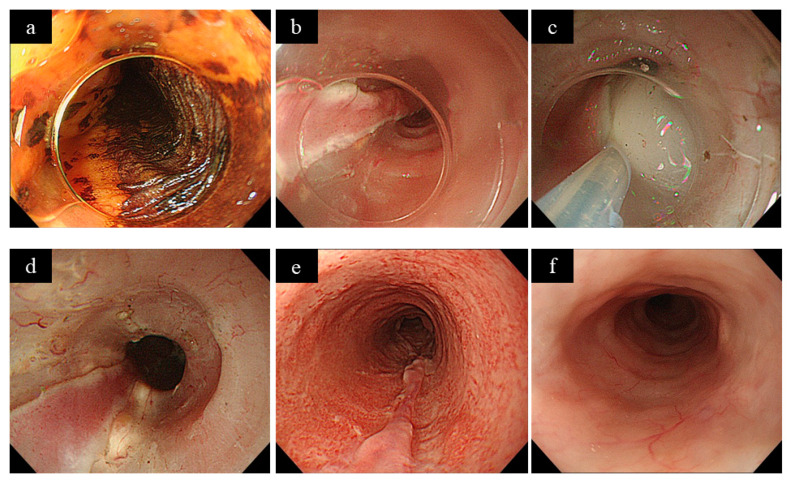
A case of steroid injection therapy. (**a**) Squamous cell carcinoma of the middle thoracic esophagus with a 3/4 circumference, 3 cm long in the long axis. (**b**) Endoscopic submucosal dissection (ESD) was performed with a mucosal defect of 7 cm in the longitudinal diameter of the 9/10th circumference. (**c**) Immediately after ESD, triamcinolone (100 mg) was administered locally to the mucosal defect. (**d**) After the injection, the injected area became white in the submucosa. (**e**) After two weeks of ESD. The mucosal defect was epithelialized. (**f**) One year after ESD. No stricture was seen.

Steroid injection has an inhibitory effect on inflammation and fibrosis, and the inhibitory effect of stricture after ESD has been demonstrated in a study using a porcine model. [47]. Among various kind of steroids, triamcinolone acetonide (TA) is commonly used for steroid injection therapy; TA is an aqueous suspension injection formulation. It is a controlled-release formulation and has the property of gradually entering the blood over a long period of time after local injection. Due to this property, the blood concentration of TA remains constant for more than three weeks after injection and remains at the injection site for more than three weeks. These effects are the reasons why TA is frequently used in injection therapy (Figure 1). Injection of other steroids, such as dexamethasone [48,49], betamethasone [33], and prednisolone [45], has also been reported, but they are absorbed rapidly.

Hashimoto et al. [21] injected TA (total dose of 18–62 mg) on days 3, 7, and 10 after ESD with more than 3/4 MDC, excluding total MDC. The incidence of stricture after TA injection was significantly lower than that in untreated patients (19% vs. 75%, respectively). Later, Hashimoto et al. [37] also reported a change in the number of TA injections from three to two, immediately after ESD (TA dose: 40–100 mg) and 14 days later (TA dose: 16–50 mg). The incidence of stricture was 45.7% among patients with more than 3/4 MDC but less than total MDC, and it was 80% among patients with total MDC. However, in the report by Funakawa et al. [50], who adopted the same method of Hashimoto’s first report regimen [21], there was no difference in the incidence of the stricture with or without TA injections among patients with more than 3/4 MDC but less than total MDC (34.8% vs. 40%, respectively). Wakahara et al. [51] conducted a randomized controlled trial (RCT) in which patients with more than 3/4 MDC, including total MDC, were treated weekly or biweekly with 40 mg of TA until the mucosal defect was closed. There was no difference in the incidence of stricture between weekly and biweekly cases (33% vs. 40%, respectively).

Currently, only one-time TA injection has become a standard injection method. Hanaoka et al. [23] first reported a one-time TA injection immediately after ESD, with a total of 100 mg. In patients with more than 3/4 MDC but less than total MDC, the incidence of stricture in patients injected with TA was significantly lower than that in treatment-free patients (10% vs. 66%, respectively). Takahashi et al. [24] conducted an RCT comparing 40 mg of TA injection immediately after ESD with no treatment in patients with tumors ranging from 2/3 to total circumference. There was no significant difference in the incidence of stricture between the two groups (62.5% and 87.5%, respectively). Nagami et al. [28] retrospectively analyzed patients with more than 2/3 MDC, excluding total MDC, using propensity score matching. The incidence of stricture was 18.9% in patients who received 80 mg of TA immediately after ESD, whereas it was 45.9% in untreated patients. Kadota et al. [27] examined the preventive effect of TA injection by the extent of each lesion according to the circumference. The incidence of stricture was 14% in patients ranging from 3/4 to less than 7/8 MDC, 56% in patients ranging from 7/8 to less than total MDC, and 100% in patients with total MDC. 

Although TA injection after esophageal ESD is beneficial, the efficacy is limited in the following cases: patients with tumors more than 3/4 circumference [34], patients with more than 5/6 MDC [36], and patients with more than 7/8 MDC [52]. Moreover, when TA is injected into the muscle layer, the risk of perforation increase [53,54], thus, a shorter needle has been developed [55].

To summarize these reports, it has become clear that TA injection has a prophylactic effect on stricture in non-total MDC. However, determining the appropriate patients for whom this treatment is effective, the appropriate dose and concentration of TA, and the appropriate site for TA injection are also issues for future research.

## 5. Oral Steroid Administration

Oral steroids are superior in that they do not require special techniques or equipment, and there is no variability in procedures, such as injection therapy. The use of oral steroids for stricture prevention after esophageal ESD was first reported by Yamaguchi et al. [16]. Prednisolone (PSL) was administered orally at a dose of 30 mg per day starting on the third day after ESD, titrated in weekly decrements of 5 mg per day, and discontinued after eight weeks. Among patients with more than 3/4 MDC, including total MDC, the incidence of stricture in patients with oral PSL was significantly lower than that in patients with prophylactic EBD (5.3% vs. 31.8%, respectively). 

Isomoto et al. [22] reported that the incidence of stricture in four patients with total MDC was 50% by Yamaguchi’s regimen [16]. Similarly, Tang et al. [56] reported that the incidence of stricture in patients with more than 3/4 of lesion circumference was 45% by Yamaguchi’s regimen. However, Zou et al. [29] reported that the incidence of stricture after 12 weeks of oral PSL was 15% among 13 patients with more than 3/4 MDC, including two patients with total MDC.

Iizuka et al. [30] reported a retrospective cohort study comparing Yamaguchi’s regimen with the modified long-term regimen. In the modified regimen, PSL was initiated at a dose of 30 mg/day and reduced by 5 mg every three weeks for 18 weeks. The incidence of stricture in patients with the modified regimen was lower than that in Yamaguchi’s regimen (36.4% vs. 82%, respectively). However, adverse events related to PSL were observed in 72.7% of patients in the modified regimen. Yamaguchi et al. [55] also extended the duration of oral PSL from eight to 18 weeks in patients with total MDC, but 33.3% of patients developed stricture. 

On the other hand, Kataoka et al. [38] reported a short-term PSL regimen. PSL was initiated at a dose of 30 mg/day and reduced in increments of one week for only three weeks. Among patients with tumors more than 3/4 circumference, including total circumferences, the incidence of stricture in patients receiving oral PSL was significantly lower than that in untreated patients (17.6% vs. 68.7%, respectively). The incidence of stricture in patients with total MDC was 33.3% with the short-term regimen.

In summary, oral steroids are as effective as injection therapy in preventing stenosis after esophageal ESD and may be more effective than injection therapy in total circumferential ESD. However, the optimal dosage and duration of oral steroids need to be considered. There are also concerns about the influences on systemic diseases, such as secondary adrenal cortical hypoplasia, hypertension, worsening of diabetes mellitus, and infection [30,57]. In our opinion, the long-term administration of oral steroids with varying doses is more complicated than injection therapy, which requires only a single session. In addition, the efficacy of oral steroids is limited even in cases of total circumferential ESD. Therefore, in the future, it is necessary to verify in which cases oral steroids are more effective than injection therapy.

## 6. Other Steroid Administration 

Mori et al. [26] reported a “steroid gel application” regimen, in which TA is mixed with jelly and administered onto the mucosal defect. They conducted an RCT among patients with more than 2/3 MDC. Patients were assigned to the combination regimen of prophylactic EBD plus steroid gel or steroid injection. In the steroid gel regimen, a mixture of 100 mg TA with jelly was sprayed onto the mucosal defect, and then EBD was performed with a 12–15 mm diameter balloon four times after 5, 8, 12, and 15 days of ESD. At 20 days post-ESD, there was no difference in the esophageal lumen diameter between the two regimens. The mean number of EBDs required in the steroid gel regimen was significantly lower than that in the steroid injection regimen (1.60 vs. 4.27, respectively).

Shibagaki et al. [42] reported the “TA-filling method,” in which a 4 mL solution of 80 mg of TA was endoscopically filled in the esophagus. The procedure was performed the day after and seven days after ESD, and endoscopies were performed every two weeks until the mucosal defect was closed. Additional procedures were performed when signs of stricture were endoscopically observed. The stricture occurred in 4.5% of patients with more than 3/4 MDC. An additional procedure was performed in 85.7% of patients with total MDC, but no stricture occurred. Later, Shibagaki et al. [43] also conducted a prospective multicenter study to evaluate the effect of the TA-filling method. Patients with more than 3/4 MDC, excluding total MDC, were included. The incidence of stricture was 5%. In addition, Kato et al. [58] reported two patients in whom the TA-filling method was used in combination with TA injection: one patient had a total MDC, and the other patient, who had 9/10 MDC, did not develop stricture. 

Sato et al. [25] reported that among patients with total MDC, patients with oral PSL plus prophylactic EBD required fewer EBDs than those with prophylactic EBD alone (13.8 times vs. 33.5 times, respectively). 

In our institution, Nakamura et al. [41] reported a systemic administration of methylprednisolone, 500 mg per day intravenously for three days as “steroid pulse therapy.” More than 3/4 MDC or longitudinal mucosal defect with more than 5 cm were included in the study. Maintenance therapy with oral PSL was not administered. It is a short-term systemic administration of steroids, a concept that completely inhibits fibroblast migration from occurring in the early stages. The incidence of stricture was 54.5%. The median number of EBDs in the stricture patients was 2.5 (range 1–6), and no adverse events were observed. 

## 7. Comparison among Steroid Therapies 

Pih et al. [32] retrospectively compared TA injection and oral PSL in patients with more than 3/4 MDC, including total MDC. Forty to 160 mg of TA injection was administered once immediately after ESD, and Yamaguchi’s regimen [16] was used for oral PSL. The incidence of stricture was 50% in untreated patients, 33.3% in TA injection, and 20% in oral PSL, and the conclusion was that oral PSL is significantly more effective than no therapy. Wang et al. [59] conducted a meta-analysis on steroid therapy and concluded that TA injection was superior to oral PSL in reducing EBD. However, the issue is that the dose and duration of the steroids are not constant in each article. 

Chu et al. [31] reported that the incidence of stricture was 14.7% in patients with more than 3/4 MDC, including total MDC, who received TA injection plus oral PSL. Eighty to 120 mg of TA was injected immediately after ESD, and oral PSL was administered according to Yamaguchi’s regimen [16]. Kadota et al. [46] reported the results of combination therapy of TA injection and oral PSL in patients with total MDC. TA had been injected at a dose of either 50 mg or 100 mg immediately after ESD, and oral PSL was administered according to Yamaguchi’s regimen. However, the incidence of stricture was 61.5%.

Furthermore, the Japan Clinical Oncology Study Group (JCOG) is now conducting an RCT to compare steroid injection therapy with the oral steroid administration in patients with non-total esophageal ESD. Hanoka’s regimen [23] is adapted as the steroid injection therapy, and Yamaguchi’s regimen [16] is adapted as the oral steroid administration. The eligible patients of this study are as follows: squamous cell carcinoma (SCC) lesions with more than 1/2 circumference but less than the total circumference and SCC lesions with less than 5 cm in longitudinal diameter [60]. The enrollment of cases has now been completed, and we are looking forward to the results of this study.

## 8. Drugs Other Than Steroids

### 8.1. Botulinum Toxin Injection Therapy

Botulinum toxin (BT) is injected into the muscle to reduce muscle contractions. In addition to reducing muscle contraction, it also has inhibitory effects on the deposition of collagen fibers and the formation of fibrous connective tissue [18].

Wen et al. [61] conducted an RCT to examine the effect of BT in patients with more than 1/2 MDC, including total MDC. Patients were assigned to receive either 100 units of BT or no drug. BT was injected immediately after ESD to reach the muscle layer. The incidence of stricture in patients with BT was significantly lower than that in patients without BT (6.1% vs. 32.4%, respectively). No serious adverse events were observed. 

BT injection is a unique and interesting method, but the procedure of injection into the muscle layer is not easy. Therefore, further validation is needed for establishing the procedure and its therapeutic effects [62].

### 8.2. Oral Tranilast

Tranilast can inhibit the release of chemical mediators from inflammatory cells and fibroblasts and directly inhibit the synthesis of collagen fibers, and has been used clinically as an anti-allergic agent and therapeutic agent for keloids.

Uno et al. [63] conducted an RCT to evaluate the additional effect of oral tranilast on prophylactic EBD with EC more than 3/4 of the circumference. Patients were assigned to a combination regimen with prophylactic EBD and tranilast (300 mg per day for eight weeks) or EBD alone. Prophylactic EBD was initiated a few days after ESD and continued for four weeks twice a week. The incidence of the stricture with the combination regimen was significantly lower than that with EBD alone (33.3% vs. 68.8%, respectively). The median number of additional EBDs was also significantly lower in the combination regimen (0 vs. four times). 

Tranilast is generally considered safer for long-term use than steroids. To pursue safer therapy, especially in patients with total MDC, the combination of oral tranilast and TA injection can be expected as the next move.

## 9. Tissue Shielding Method

### 9.1. Polyglycolic Acid Sheet

**Figure 2:**
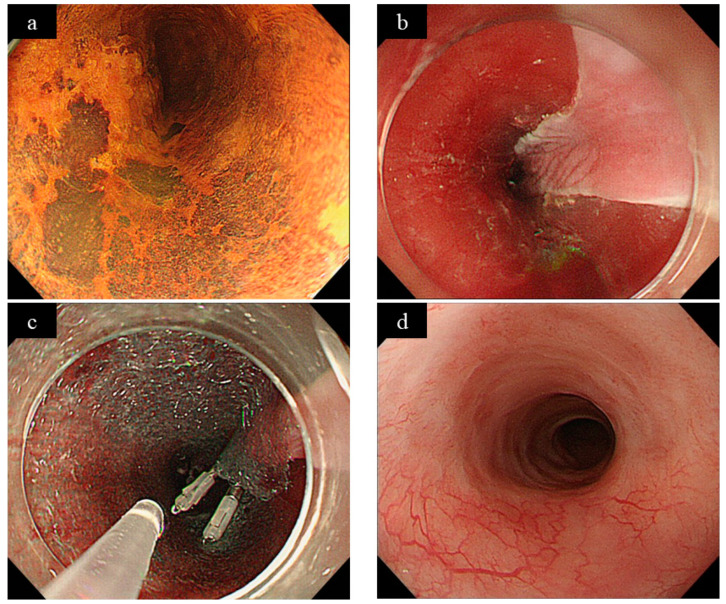
A case of polyglycolic acid (PGA) sheet application (courtesy of Dr. Ono of the University of Tokyo). (**a**) Squamous cell carcinoma of the middle thoracic esophagus with a circumference of more than 1/2. (**b**) Endoscopic submucosal dissection (ESD) was performed, resulting in an approximately 5/6 circumferential mucosal defect. (**c**) The PGA sheet was coated over the mucosal defect by the clip and pull method. (**d**) Six months after ESD. The mucosal defect was completely epithelialized, and no stricture occurred.

Polyglycolic acid (PGA) sheets have been used in combination with fibrin glue to cover wounds. Iizuka et al. [64] reported that in patients with more than 1/2 MDC, excluding total MDC, multiple cut PGA sheets were applied to the mucosal defect immediately after ESD. The incidence of stricture after six weeks was 7.7%. Iizuka et al. [35] also reported that the incidence of stricture in PGA sheet patients was comparable to that in TA injection patients (9.1% vs. 10.3%, respectively). Ono et al. [65,66] devised a “clip and pull method”, in which the PGA sheet is clipped to the esophageal mucosa in one piece without being cut into small pieces (Figure 2). Sakaguchi et al. [67] reported that the incidence of stricture was 37.5% by the “clip and pull method” on more than 3/4 MDC. 

Judging that the PGA sheet alone was an insufficient effect to prevent stricture, Sakaguchi et al. [40] combined the PGA sheet with TA injection (40 mg). The incidence of stricture was 11.1% in non-total MDC and 50% in total MDC. Later, Sakaguchi et al. [44] also reported in an analysis of 349 consecutive patients treated for stricture prevention that combining the PGA sheet with TA injection (40 mg) had a lower stricture rate than the PGA sheet alone (18.9% vs. 41.4%), when cervical esophageal lesions and non-total MDCs, which are strong independent risk factors for stricture, were excluded. In addition, Nagami et al. [39] combined the PGA sheet with TA injection (80 mg) in patients with more than 5/6 MDC, and the incidence of stricture was 25% in non-total MDC and 66.7% in total MDC. Based on these two studies, the PGA sheet and TA injection may be one of the options for stricture prevention of total MDC, but they are not fully effective.

### 9.2. Carboxymethyl Cellulose Sheet

Carboxymethyl cellulose (CMC) has been reported as an injectant for gastric ESD [68,69]. Lua et al. [70] covered the post-ESD mucosal defect with a CMC sheet in patients with one or more of the following three conditions: cervical esophagus, tumor circumference greater than 1/2, and tumor longitudinal length greater than 4 cm. The incidence of stricture was 57%. Tang et al. [71] performed a basic study in pigs. The incidence of stricture seven days after ESD was 71.4% in the CMC sheet group and 100% in the treatment-free group. From these results, the CMC sheet alone is insufficient to prevent the stricture of ESD.

## 10. Regenerative Medicine

The application of regenerative medicine has been studied mainly in animals to prevent stricture after ER [72,73,74,75,76,77,78,79,80,81,82,83,84,85,86]. Transplantation of autologous oral mucosal epithelial cell (AOMEC) sheets have been developed particularly, although auto gastrointestinal transplantation involving the gastric mucosa [87] and esophageal mucosa [88] failed to show sufficient efficacy.

**Figure 3:**
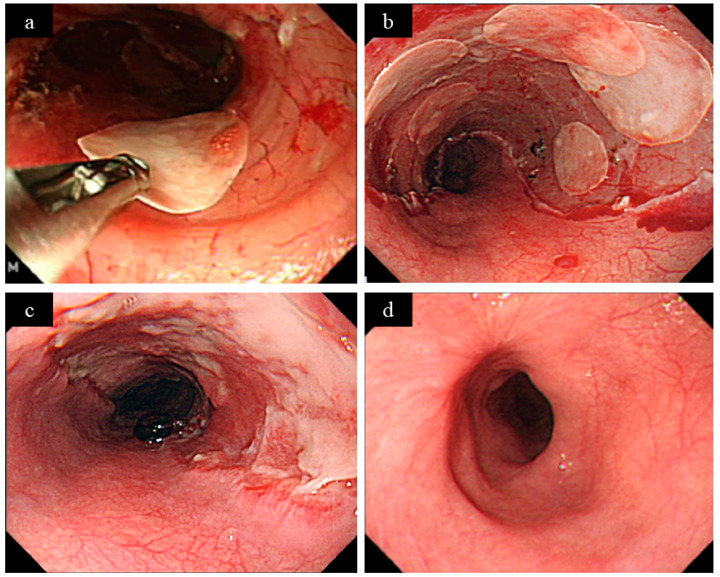
A case of autologous oral mucosal sheet transplantation (courtesy of Dr. Ohki of Tokyo Women’s Medical University). (**a**) Oral mucosal sheets were implanted in the mucosal defect after endoscopic submucosal dissection (ESD) using grasping forceps. (**b**) Transplantation with seven oral mucosal sheets was performed. (**c**) One week after ESD, epithelialization was observed in the mucosal defect. (**d**) After three weeks of ESD, the mucosal defect was almost epithelialized.

Ohki et al. [89] focused on AOMECs and reported them in a canine model. They successfully adhered to the mucosal defect after esophageal ESD, promoting wound healing and preventing esophageal stricture. Subsequently, Kanai et al. [90] demonstrated that AOMEC sheet transplantation prevented stricture in pigs with total circumferential ESD. Murakami et al. [91] and Takagi et al. [92,93] developed a new tissue-engineered cell sheet of human origin, and then Ohki et al. [94,95] applied this clinically (Figure 3). In patients with more than 1/2 MDC by ER, AOMEC sheets completely epithelialized mucosal defects at a median of three weeks. The incidence of stricture was 10%. They have also succeeded in developing a logistics system and new devices to collect materials from clinics, transport them to the remote cell proceeding center, and return the cultured AOMECs for endoscopic transplantation [96,97,98,99].

Regarding the issue, AOMEC sheets have high manufacturing costs and cannot be easily implemented in any facility. In addition, due to the limited amount of oral mucosa that can be harvested, it appears that AOMEC sheets of sufficient size for treating extensive mucosal defects cannot be cultured. However, clinical trials are currently underway, and we look forward to future developments.

## 11. Stent Placement

Stent placement for stricture formation after ER for esophageal cancer has been reported [100,101]. On the other hand, regarding prevention, Wen et al. [102] performed an RCT with more than 3/4 MDC, including total MDC. After 12 weeks of ESD, the incidence of stricture in the stent group was significantly lower than that in the non-stent group (18.2% vs. 72.7%, respectively). Ye et al. [103] placed a 16–18 mm diameter full-covered metal stent 12 weeks immediately after ESD in patients with total MDC. The incidence of stricture was 17.4%. 

Chai et al. [104] performed an RCT with patients with more than 3/4 MDC, including total MDC, to compare stents covered with PGA sheets and stents alone. A 17 mm diameter stent was placed immediately after ESD, and the stent covered by the PGA sheet was removed at four weeks and the stent alone at eight weeks. The incidence of stricture in the PGA sheet-covered stent was significantly lower than that in the stent alone (20.5% vs. 46.9%, respectively). Li et al. [105] further studied stents covered by PGA sheets soaked with TA and placed them in patients with more than 3/4 MDC, including total MDC; 17 mm full-coverage metal stents covered with PGA sheets were soaked with 80 mg of TA diluted with saline on the PGA sheets. The stent was placed for 4–6 weeks immediately after ESD. The incidence of stricture was 100% and 50% in patients with more than 3/4 MDC and with total MDC, respectively.

Although stenting is a simple procedure and is presumed to be highly effective in mechanically reducing stricture, stent migration and perforation are of concern. Cautions for the appropriate stent placement site, length of the stent, and timing of placement are required. Metallic stents may be limited to the treatment of contraindications to oral steroids [106].

Recently, biodegradable stents have been used for refractory benign esophageal strictures. Saito et al. [107] and Yano et al. [108] reported a small number of patients for treating ESD stricture, whereas Saito et al. [109] reported the prevention of stricture after ESD. Biodegradable stents were placed 2–3 days after ESD in seven patients with more than 3/4 MDC. No stricture occurred.

## 12. Conclusions

Steroid therapy is the current mainstay of stricture prevention after esophageal ESD, although it is not clear whether TA is more effective than oral PSL. Focuses have shifted to ways to prevent stricture after total MDC, where TA injection plus oral PS or steroid therapy plus tissue shielding has been attempted. It is expected that AOMEC sheet transplantation and biodegradable stent implantation will be widely applied in the future. 

## Figures and Tables

**Table 1:** Prevention of stricture after endoscopic submucosal dissection for esophageal for esophageal squamous cell carcinoma.

Prophylactic EBD	
Steroid therapy	Steroid injection therapy (ex. TA)
	Oral steroid administration (ex. PSL)
	Other steroid administration: combination of TA injection with oral PSL, TA injection with PGA, TA injection with EBD, TA-filling method
Drugs other than steroids	Botulinum toxin injection therapy
	Oral tranilast
Tissue shielding method	PGA sheet
	Carboxymethyl cellulose sheet
Regenerative medicine	Autologous oral mucosal epithelial cell sheet transplantation, et al.
Stent placement	Temporary metal stent placement, bioabsorbable stent placement

EBD, endoscopic balloon dilation; TA, triamcinolone acetate; PSL, prednisolone; PGA, polyglycolic acid.

**Table 2:** Comparative studies of steroid therapy in the prevention of stricture after endoscopic submucosal dissection for esophageal squamous cell carcinoma, mainly compared with no therapy or prophylactic endoscopic balloon dilation.

Author	Year	Study Design	Protocol Therapy	Mucosal Deffect Circumference	Case Numbers (Protocol: Control)	Incidence of Stricture (Protocol vs. Control)	*p*-Value *^1^
Hashimoto [21]	2011	Retrospective, historical control	TA injection	>3/4	21:20 (untreated)	19% vs. 75%	<0.001
Yamaguchi [16] *^2^	2011	Retrospective, historical control	Oral PSL for eight weeks	>3/4	19:22 (prophylactic EBD) *^3^	5.3% vs. 31.8%	0.03
Isomoto [22] *^2^	2011	Retrospective, historical control	Oral PSL for eight weeks	Total circumference	4:3 (prophylactic EBD)	50% vs. 100%	N.S.
Hanaoka [23]	2012	Prospective, historical control	TA injection	>3/4	30:29 (untreated)	10% vs. 66%	<0.001
Takahashi [24]	2012	Prospective, randomized	TA injection	Lesion > 2/3	16:16 (untreated) *^4^	62.5% vs. 87.5%	0.22
Sato [25]	2013	Prospective, historical control	Oral PSL for eight weeks + prophylactic EBD	Total circumference	10:13 (prophylactic EBD) *^5^	100% vs. 100%	N.S.
Mori [26]	2013	Prospective, randomized	① TA gel + prophylactic EBD ② TA injection + prophylactic EBD	>2/3	20:21 (①:②)	N/A *^6^	N/A
Kadota [27]	2016	Retrospective	① TA injection + Oral PSL for eight weeks ② TA injection	>3/4	29:53:33 (①:②: untreated)	41% vs. 43% Vs. 67% (①:②: untreated)	0.073 (① vs. untreated) 0.046 (② vs. untreated)
Nagami [28]	2017	Retrospective, matched	TA injection	>2/3	37:37 (untreated)	18.9% vs. 45.9%	0.016
Zhou [29]	2017	Retrospective	Oral PSL for 12 weeks	>3/4 *^7^	13:10 (untreated)	23.1% vs. 80%	<0.05
Iizuka [30]	2018	Retrospective, historical control	① Oral PSL for 18 weeks (±TA injection) *^8^ ② Oral PSL for eight weeks (±TA injection) *^8^	Total circumference	11:11 (①:②)	36.4% vs. 82%	0.04
Chu [31]	2019	Retrospective	TA injection + Oral PSL for eight weeks	>2/3	34:36 (untreated)	14.7% vs. 52.8%	0.001
Pih [32]	2019	Retrospective	① Oral PSL ② TA injection	>3/4	25:6:22 (①: ②: untreated)	20% vs. 33.3% vs. 50% (①:②: untreated)	0.037 (① vs. untreated) 0.046 (①+② vs. untreated)

TA, triamcinolone acetate; PSL, prednisolone; EBD, endoscopic balloon dilation; N/A, not available; NS, not significant. *1: *p*-values are presented as described in the literature. *2: Yamaguchi and Isomoto belong to the same institution. *3: Among them, three cases have the total circumferential mucosal defect. *4: Among them, 11 cases have mucosal defect circumference > 3/4. *5: Among them, one case of protocol therapy was adenocarcinoma. *6: The definition of stricture rate is different from that reported in other literature. *7: Among them, two cases have the total circumferential mucosal defect. *8: TA injections were performed in 10 cases in Group 1 and six cases in Group 2.

**Table 3:** Effect of preventive steroid therapy after non-total circumferential endoscopic submucosal dissection for esophageal squamous cell carcinoma.

Author	Year	Study Design	Drugs	Dose	Timing of Intervention	Mucosal Defect Circumference	Incidence of Stricture
	Steroid injection						
Hashimoto [21]	2011	Retrospective	TA	18–62 mg	Day 3, 7, 10 (3 times)	>3/4	19% (4/21)
Hanaoka [23]	2012	Prospective	TA	100 mg	Day 0	>3/4	10% (3/30)
Yamaguchi [33]	2013	Retrospective	TA	40 mg (<3 cm in longitudinal mucosal defect), 80 mg (≥ 3 cm)	Day 0 (>9/10 in circumference or ≥5 cm in longitudinal mucosal defect: additionally Day 21)	>3/4	4.3% (1/23)
Takahashi [24]	2015	Prospective, randomized	TA	40 mg	Day 0	>2/3 (lesion *)	45.5% (5/11)
Hanaoka [34]	2016	Retrospective	TA	50–100 mg	Day 0	>3/4	11.3% (13/115)
Kadota [27]	2016	Retrospective	TA	50 mg	Day 3, 7, 10 (three times) →Day 1 or Day 0 (once)	>3/4	36.2% (17/47)
Nagami [28]	2017	Retrospective	TA	80 mg	Day 0	>2/3	20.7% (12/58)
Iizuka [35]	2017	Retrospective	TA	40 mg	Day 0	>1/2	10.3% (3/29)
Nagami [36]	2018	Retrospective	TA	80 mg	Day 0	>2/3	16.8% (17/101)
Hashimoto [37]	2019	Retrospective	TA	40–100 mg (2nd session: 16–50 mg)	Day 0, 14 (two times)	>3/4	45.7% (16/35)
	Oral steroid administration						
Yamaguchi [16]	2011	Retrospective	PSL	30 mg	Tapering gradually for eight weeks	>3/4	6.3% (1/16)
Yamaguchi [33]	2013	Retrospective	PSL	30 mg	Tapering gradually for 6–12 weeks	>3/4	10% (4/40)
Kataoka [38]	2015	Retrospective	PSL	30 mg	Tapering gradually for three weeks	>3/4	14.3% (2/14)
	Modified or hybrid steroid therapy						
Kadota [27]	2016	Retrospective	TA + Oral PSL	TA: 50 mg PSL: 30 mg	TA: Day 3, 7, 10 (three times) →Day 1 or Day 0 (once) PSL: tapering gradually for eight weeks	>3/4	13.3% (2/15)
Nagami [39]	2016	Retrospective	TA injection + PGA	TA: 80 mg	Day 0	>5/6	25% (1/4)
Sakaguchi [40]	2016	Retrospective	TA injection + PGA	TA: 40 mg	Day 0	>3/4	11.1% (1/9)
Nakamura [41]	2017	Prospective	Pulse therapy	mPSL: 500 mg (intravenous administration)	Day 1, 2, 3 (three consecutive days)	>3/4	66.7% (6/9)
Shibagaki [42]	2018	Retrospective	TA filling method	TA: 80 mg	Day 1 and Day 7 and when mild stricture was found	>3/4	6.7% (1/15)
Shibagaki [43]	2020	Prospective	TA filling method	TA: 80 mg	Day 1 and Day 7 and when mild stricture was found	>3/4	5% (1/20)
Sakaguchi [44]	2020	Retrospective	TA injection + PGA	TA: 40 mg	Day 0	>3/4	18.9% (7/37)

TA, triamcinolone acetonide; PSL, prednisolone; PGA, polyglycolic acid; mPSL, methylprednisolone. The dose was shown in one session. Day 0 means immediately after ESD. * Lesion circumference (not mucosal defect circumference).

**Table 4:** Effect of preventive steroid therapy after total circumferential endoscopic submucosal dissection for esophageal squamous cell carcinoma.

Author	Year	Study Design	Drugs	Dose	Timing of Intervention	Incidence of Stricture
	Steroid injection					
Yamaguchi [33]	2013	Retrospective	TA	80 mg	Day 0, 21	100% (4/4)
Takahashi [24]	2015	Prospective, randomized	TA	40 mg	Day 0	100% (5/5)
Hanaoka [34]	2016	Retrospective	TA	100 mg	Day 0	91.7% (11/12)
Miwata [45]	2016	Retrospective	PSL	N/A	Day 1	100% (6/6)
Hashimoto [37]	2019	Retrospective	TA	40–100 mg (second: 16–50 mg)	Day 0, 14 (two times)	80% (4/5)
	Oral steroid administration					
Yamaguchi [16]	2011	Retrospective	PSL	30 mg	Tapering gradually for eight weeks	0% (0/3)
Isomoto [22]	2011	Retrospective	PSL	30 mg	Tapering gradually for eight weeks	50% (2/4)
Sato [25]	2013	Prospective	PSL	30 mg	Tapering gradually for eight weeks	100% (10/10)
Yamaguchi [33]	2013	Retrospective	PSL	30 mg	Tapering gradually for 8–18 weeks	27.3% (3/11)
Kataoka [38]	2015	Retrospective	PSL	30 mg	Tapering gradually for three weeks	33.3% (1/3)
Miwata [45]	2016	Retrospective	PSL	0.5 mg/kg	Tapering gradually 5 mg/week	100% (13/13)
	Modified or hybrid steroid therapy					
Kadota [27]	2016	Retrospective	TA + Oral PSL	TA: 50 mg PSL: 30 mg	TA: Day 3, 7, 10 (three times) →Day 1 or Day 0 (once) PSL: tapering gradually for eight weeks	71% (10/14)
Nagami [39]	2016	Retrospective	TA injection + PGA	TA: 80 mg	Day 0	66.7% (4/6)
Sakaguchi [40]	2016	Retrospective	TA injection + PGA	TA: 40 mg	Day 0	50% (1/2)
Iizuka [30]	2018	Retrospective	Oral PSL ±TA injection	PSL: 30 mg TA: 80–120 mg	PSL: tapering gradually for eight weeks (TA injection: Day 0)	81.8% (9/11)
			Oral PSL ±TA injection	PSL: 30 mg TA: 80–120 mg	PSL: tapering gradually for 18 weeks (TA injection: Day 0)	36.4% (4/11)
Shibagaki [42]	2018	Retrospective	TA filling method	TA: 80 mg	Day 1 and Day 7 and when mild stricture was found	0% (0/7)
Kadota [46]	2020	Retrospective	TA + Oral PSL	TA: 50 or 100 mg PSL: 30 mg	TA: Day 0 PSL: tapering gradually for eight weeks	61.5% (16/26)

TA, triamcinolone acetonide; PSL, prednisolone; PGA, polyglycolic acid; N/A, not available. The dose was shown in one session. Yamaguchi and Isomoto belong to the same institution.
